# An Ancient Lineage of Highly Divergent Parvoviruses Infects both Vertebrate and Invertebrate Hosts

**DOI:** 10.3390/v11060525

**Published:** 2019-06-06

**Authors:** Judit J. Pénzes, William Marciel de Souza, Mavis Agbandje-McKenna, Robert J. Gifford

**Affiliations:** 1 McKnight Brain Institute and Department of Biochemistry and Molecular Biology, University of Florida, 1149 Newell Dr, Gainesville, FL 32610, USA; mckenna@ufl.edu; 2 Virology Research Center, School of Medicine of Ribeirão Preto of the University of São Paulo, Ribeirão Preto, Brazil; wmarciel2@gmail.com; 3 Medical Research Council-University of Glasgow Centre for Virus Research, 464 Bearsden Road, Glasgow G61 1QH, UK

**Keywords:** chapparvovirus, parvovirus evolution, endogenous viral elements, *Parvoviridae*, densovirus, homology modeling, new viruses

## Abstract

Chapparvoviruses (ChPVs) comprise a divergent, recently identified group of parvoviruses (family *Parvoviridae*), associated with nephropathy in immunocompromised laboratory mice and with prevalence in deep sequencing results of livestock showing diarrhea. Here, we investigate the biological and evolutionary characteristics of ChPVs via comparative in silico analyses, incorporating sequences derived from endogenous parvoviral elements (EPVs) as well as exogenous parvoviruses. We show that ChPVs are an ancient lineage within the *Parvoviridae*, clustering separately from members of both currently established subfamilies. Consistent with this, they exhibit a number of characteristic features, including several putative auxiliary protein-encoding genes, and capsid proteins with no sequence-level homology to those of other parvoviruses. Homology modeling indicates the absence of a β-A strand, normally part of the luminal side of the parvoviral capsid protein core. Our findings demonstrate that the ChPV lineage infects an exceptionally broad range of host species, including both vertebrates and invertebrates. Furthermore, we observe that ChPVs found in fish are more closely related to those from invertebrates than they are to those of amniote vertebrates. This suggests that transmission between distantly related host species may have occurred in the past and that the *Parvoviridae* family can no longer be divided based on host affiliation.

## 1. Introduction

Parvoviruses (family *Parvoviridae*) are small, non-enveloped viruses with T = 1 icosahedral symmetry and linear, single-stranded DNA (ssDNA) genomes ~4–6 kilobases (kb) in length. The family has historically been divided into two subfamilies, *Parvovirinae* and *Densovirinae*, containing viruses that infect vertebrate and invertebrate hosts, respectively [1]. Despite exhibiting great variation in expression and transcription strategies, they have a relatively conserved overall genome structure: a non-structural (NS) expression cassette is located at the left side of the genome, while the structural viral proteins (VPs) are encoded by the right, and complex, hairpin-like DNA secondary structures are present at both genomic termini [2]. Small satellite proteins and an assembly-activating protein have been discovered as products of open reading frames (ORFs) overlapping the right-hand expression cassette, whereas additional auxiliary protein-encoding ORFs may be positioned between the two major cassettes [3,4]. 

Numerous novel parvoviruses have been identified in recent years, primarily via approaches based on high throughput sequencing (HTS) [5,6,7,8,9,10,11]. In addition, progress in whole genome sequencing of eukaryotes has revealed that sequences derived from parvoviruses occur relatively frequently in animal genomes [12,13,14,15,16,17]. These endogenous parvoviral elements (EPVs) are derived from the genomes of ancient parvoviruses that were incorporated into the gene pool of ancestral host species. This can presumably occur when infection of a germline cell leads to parvovirus-derived DNA becoming integrated into host chromosomes, and the cell containing the integrated sequences then goes on to develop into a viable organism [18]. Many EPVs are millions of years old, and are genetically “fixed” in the genomes of host species (i.e., all members of the species have the integrated EPV in their genomes). Such ancient EPV sequences are in some ways analogous to “parvovirus fossils”, since they preserve information about the ancient parvoviruses that infected ancestral animals.

Among the novel parvovirus groups identified via sequencing, one—provisionally labeled “chapparvovirus”—stands out as being particularly unusual. These viruses, which have been primarily reported via metagenomic sequencing of animal feces, derive their name from an acronym (CHAP), referring to the host groups in which they were first identified (Chiropteran–Avian–Porcine) [15,16,19,20]. Subsequently, several additional chapparvovirus (ChPV) sequences have been reported, including some that were identified in whole genome sequence (WGS) data derived from vertebrates, including reptiles, mammals, and birds [9]. These sequences were picked up by in silico screens designed to detect EPVs. However, since all the ChPV sequences identified in WGS data lack clear evidence of genomic integration, it is likely that they actually derive from infectious ChPV genomes that contaminated WGS samples, rather than from endogenous elements [9].

Until relatively recently, evidence that the ChPVs detected via sequencing actually infected vertebrate hosts has been lacking. However, a recent study has claimed to demonstrate that a ChPV called mouse kidney parvovirus (MKPV) circulates among laboratory mice populations, in which it causes a kidney disease known as inclusion body nephropathy [21]. These findings, as well as their frequent presence in the feces of livestock, imply that ChPVs might be pathogenic and represent a potential disease threat to wildlife and domestic species. In addition, they have raised interest in the use of these viruses as experimental tools. In this study, we perform a comparative analysis of ChPV genomes and ChPV-derived EPVs, revealing new insights into the biology and evolution of this poorly understood group.

## 2. Materials and Methods

### 2.1. Genome Screening and Sequence Analysis

All WGS data were obtained from the National Center for Biotechnology Information (NCBI) genomes resource. We obtained all available genomes for eumetazoan animals as of October 2018. These data were screened for ChPV sequences using the database-integrated genome screening (DIGS) tool [22]. ChPV sequences were characterized and annotated using Artemis Genome Browser [23]. The NCBI Basic Local Alignment Search Tool (BLAST) program and its local executables were used to compare sequences and investigate predicted viral ORFs. To determine potential homology and sequence similarity, even between previously undescribed ORFs, we constructed a local database, including all ORFs exceeding 100 amino acids (aa) in length, derived from all the exogenous and endogenous sequences incorporated in this study, and used the local BLAST P and X algorithms to conduct similarity searches in it. Two ORFs were accepted as homologous if they gave a significant hit, in the case of an expectation value threshold of 1.

Promoters were predicted using the neural network-based promoter prediction server of the Berkeley Drosophila Genome Project and further verified by the Promoter Prediction 2.0 server [24,25]. Splice sites were also detected using the neural network-based applications of the Berkeley Drosophila Genome project and SplicePort [25,26]. Polyadenylation signals were predicted by the SoftBerry application POLYAH [27]. To verify that these applications were be capable of detecting the above-mentioned chapparvoviral transcription elements we ran MKPV through the workflow pipeline.

### 2.2. Phylogenetic Reconstructions

The derived aa sequences of ORFs disclosing homology to parvoviral NS1 proteins were aligned with at least five representatives of each parvovirus genus, or with one representative of each species of a given genus in cases where the number of species did not exceed five. To ensure the correct identification of the tripartite helicase domain, structural data was also incorporated into alignment construction using T-coffee Expresso [28] and Muscle [29]. The full-length NS1 derived aa sequences of the ChPV clade were aligned by Muscle and the M-coffee algorithm of T-coffee [30]. Model selection was carried out by ProTest and the substitution models RtREV+I+G, in cases of helicase-based inferences, and LG+I+G, for the complete chapparvoviral NS1 tree, were predicted to be the most suitable, based on both Akaike and Bayes information criteria. The PhyML-3.1 program was used to infer a maximum likelihood phylogenetic tree, with 100 bootstrap iterations [31], based on a guide tree previously constructed by the ProtDist and Fitch programs of the Phylip 3.697 package [32].

### 2.3. Homology Modeling and DNA Structure Prediction

Structural homology was detected by applying the pGenTHREADER and pDomTHREADER algorithms of the PSIPRED Protein Sequence Analysis Workbench [33]. The same workbench was used to map disordered regions using DISOPRED3 and to predict the secondary structure of the complete chapparvoviral VP protein sequences via the PSIPRED algorithm. The selected PDB structures were applied as templates for homology modeling, carried out by the I-TASSER Standalone Package v.5.1 [34]. To guide the modeling, the predicted secondary structures were applied as a restriction. The Oligomer Generator feature of the Viper web database (http://viperdb.scripps.edu/) [35] was used to construct 60-mers of the acquired putative VP monomer structures. Surface images of the capsids were rendered using the PyMOL Molecular Graphics System [36]. Capsid surface maps and VP monomer superposition were carried out by UCSF Chimera [37]. To predict the presence of potential DNA secondary structural elements, the DNA Folding Form algorithm of the mFold web server was utilized [38].

## 3. Results

### 3.1. Comparative Analysis of Previously Reported ChPV Genomes

**Figure 1:**
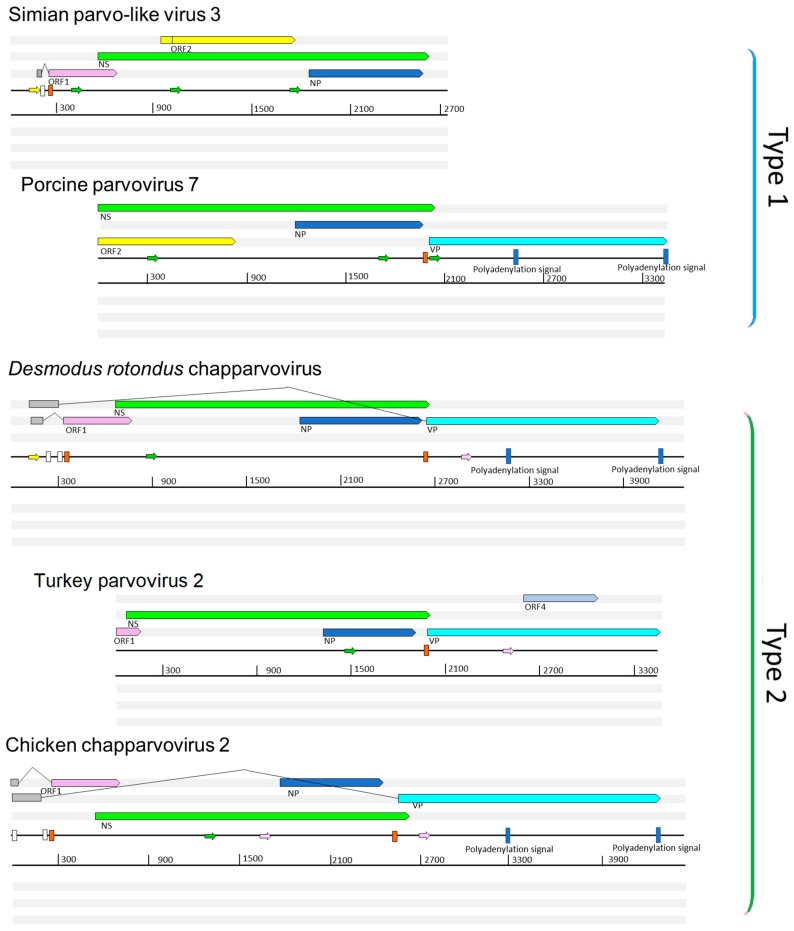
Representative complete coding sequence and partial genome organizations of the two distinct types of exogenous amniote chapparvoviruses (ChPVs). Open reading frames (ORFs) are represented by rectangular arrows, colored according to homology. Splice donor sites are marked by white-colored bars, acceptor sites by orange-colored bars. Blue-colored bars show predicted polyadenylation signals. Small arrows show predicted promoters and are colored according to prediction score (>0.95 = green; 0.9–0.95 = pink: <0.9 = yellow). Grey boxes indicate regions inferred to be transcribed but not translated. Note: ORF4 is unique to turkey parvovirus and is not found in other avian type 2 ChPVs, such as chicken ChPV2.

We performed a comparative analysis of nine previously sequenced ChPV genomes so that we could: (i) identify genome features that characterize these viruses, and (ii) make inferences about aspects of ChPV biology and evolution (Figure 1). ChPV genomes tend toward the shorter end of the parvovirus genome size range (~4 kb). They encode a relatively long *rep* gene, and a relatively short *cap* gene. The *rep* gene product (NS) is ~650 amino acids (aa) in length, with the longest example being the 668 aa protein encoded by *Desmodus rotondus* ChPV (DrChPV). ChPV NS proteins contain ATPase and helicase domains, but these are the only regions exhibiting clear homology to those found in other parvovirus groups (Figure 1). Overlapping the *rep*, a predicted minor ORF, ~220 aa in length, is located in a position equivalent to that of the nucleoprotein (NP) ORF found in certain *Parvovirinae* genera (i.e., *Ave*- and *Bocaparvovirus*). However, it should be noted that the protein encoded by this gene—which we tentatively refer to as NP—exhibits no significant similarity to any other parvovirus NP proteins. Secondary structure predictions indicate that the vast majority of the NP protein has a helical structure, with numerous potential phosphorylation sites as well as a potentially protein-binding disordered N-terminus (Figure S1). Together, these observations suggest a non-structural function. The NP ORF, although of similar length in all genomes, has no canonical start codon in the case of porcine parvovirus 7 (PPV7) and simian parvo-like virus 3 (SiPV3). This would imply that in these viruses, splicing of the *rep* RNA is required for expression of the NP protein.

All ChPVs appear to be characterized by relatively short VP ORFs of 450–500 aa. VP proteins are typically ~650–820 aa in most other parvoviruses, the exception being the brevi- and penstyldensoviruses, which encode an even shorter VP. Notably, the VP proteins encoded by ChPVs share no significant sequence similarity with those of other parvoviruses. In all ChPVs, the first methionine of the VP ORF is preceded by a potential coding sequence, and in all published ChPV sequences, a canonical splice acceptor site is located immediately upstream. Possibly, the VP ORF encodes only the major capsid protein, and there may be other versions of this VP protein that are elongated at the N-terminus, and are incorporated into the capsid at a lower copy number, as found in the majority of parvoviruses [1]. However, the only splice donor sites we identified are located relatively far upstream. In MKPV, however, there are two large introns present, putting these upstream exons in frame with the VP encoding exon.

In addition to their fundamental NS–NP–VP genome organization, ChPVs encode various additional small ORFs. ORF1 is predicted to encode a small protein of approximately 15 kDa that contains a putative nuclear localization signal (NLS) in its C-terminal region. ORF1, which partially overlaps the N-terminal region of NS, is present in all genomes except PPV7. However, since the PPV7 genome also lacks the corresponding region of NS, this likely reflects a 5’ truncated genome sequence. The same is the case for turkey parvovirus (TPV2), although the C-terminal encoding region of the putative ORF1 protein could be revealed.

A second additional, putative ORF is present in only two of the ChPVs examined here: PPV7 and simian parvo-like virus 3. This ORF, referred to as ORF2, is located downstream from ORF1 in a position completely overlapping the NS ORF. The TPV2 genome also contains a unique, presumably genome-specific additional ORF (ORF4) that overlaps the C-term encoding region of VP, and may encode a predicted 17 kDa protein (Figure 1). Interestingly, this ORF was absent from the other, closely-related avian ChPVs.

Analysis in silico revealed at least three potential promoters in ChPV genomes. One of these is conserved throughout the clade, and is located upstream of all coding features, indicating that it likely drives early expression of virus genes. Moreover, its presence has been confirmed in MKPV by sequencing of cDNA derived from infected mouse tissue. None of the other potential promoters proved to be functional in the case of MKPV. The MKPV transcriptome includes three transcripts confirmed to undergo splicing. Of these, however, only the one with the shortest intron could be confidently predicted in all GenBank sequences with a complete or near complete coding region (Figure 1). Interestingly, DrChPV (similar to rodent-derived ChPVs) and chicken ChPV2 (similar to TPV2, but with a more complete 5’ end) were both predicted to possess the large intron of the putative VP transcript, and therefore appear to utilize a strikingly MKPV-like transcription mechanism, despite missing an acceptor site upstream of the NP start codon. In all ChPVs examined, with the exception of the 3’ truncated entries, we identified two potential polyadenylation signals in positions equivalent to those found in MKPV [21]. This implies that the polyadenylation strategy is a conserved feature of ChPV transcription.

### 3.2. Identification and Characterization of Novel ChPVs and ChPV-Derived EPVs

**Figure 2:**
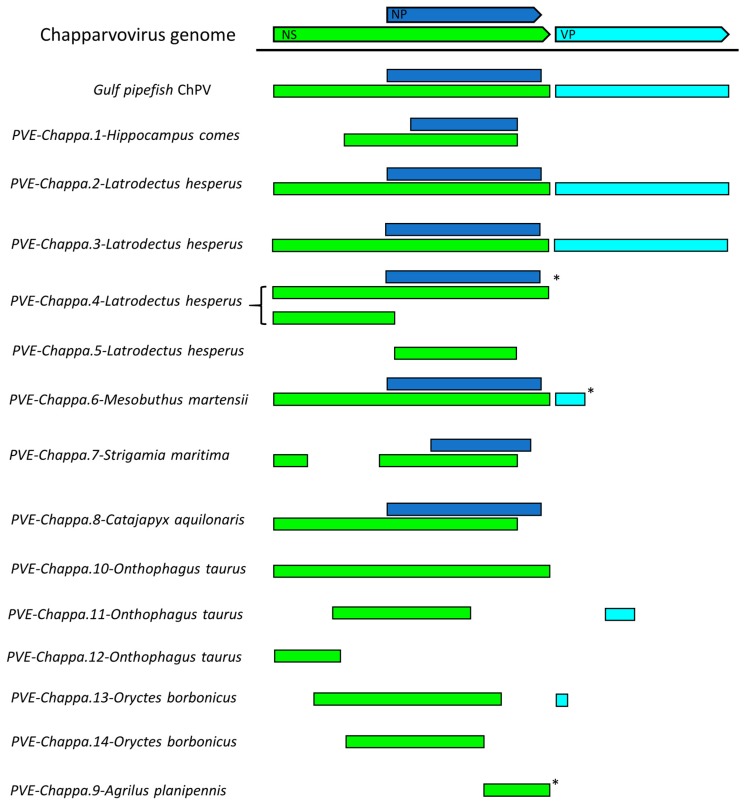
Basic gene content of newly identified chapparvoviruses (ChPVs) and ChPV-derived endogenous parvoviral element (EPV) sequences, shown in relation to a representative ChPV genome (mouse kidney parvovirus). Asterisks indicate contigs that were truncated within the virus-derived portion of the sequence. Abbreviations: non-structural protein (NS); capsid protein (VP); nucleoprotein (NP).

We systematically screened published WGS data and identified a total of 15 previously unreported ChPV-derived DNA sequences. Two were identified in vertebrates and 13 in invertebrates (Table 1). The majority of the novel ChPV sequences identified in our screen were derived from the non-structural protein gene (*rep*), but we identified complete sequences derived from both the *rep* and capsid (*cap*) genes in two species: the Gulf pipefish (*Syngnathus scovelli*) and the black widow spider (*Latrodectus hesperus*). Partial *cap* genes were identified in the scarab beetle (*Oryctes borbonicus*), taurus scarab (*Onthophagus taurus*), and Chinese golden scorpion (*Mesobuthus martensii*) elements (Figure 2).

We identified two chapparvoviral sequences in WGS assemblies of syngnathid fish (family Syngnathidae), including the tiger tail seahorse (*Hippocampus comes*) and the Gulf pipefish (*Syngnathus scovelli*). The pipefish sequence occurs in a relatively short scaffold (4002 nt) that is entirely comprised of viral sequence, displaying truncated, but nonetheless detectable, J-shaped terminal hairpin-like structures (Figure S2). This suggests it likely represents a virus contaminant, as suspected for other ChPV sequences recovered from vertebrate WGS data [9]. The virus from which this sequence was presumably derived was designated *Syngnathus scovelli* ChPV (ScChPV). 

The seahorse and invertebrate sequences identified in our screen clearly represented EPVs (see below). However, the pipefish sequence lacked flanking genomic sequences and appeared to derive from an exogenous virus, encompassed by truncated hairpin-like secondary structure repeats (Figure S2). None of the ChPV-derived EPVs we identified shared homologous flanking sequences, indicating that each derives from a distinct germline incorporation event.

We identified a total of 13 EPV sequences that disclosed a relatively close phylogenetic relationship to ChPVs. These elements showed varying degrees of degradation. In many cases, only genome fragments were detected (Figure 2), and these usually included multiple nonsense mutations (Table 1). ChPV-derived elements were detected in three major arthropod clades that primarily occupy terrestrial habitats, namely arachnids of Chelicerata, chilopods of Myriapoda, as well as hexapod insects and entognaths.

**Figure 3:**
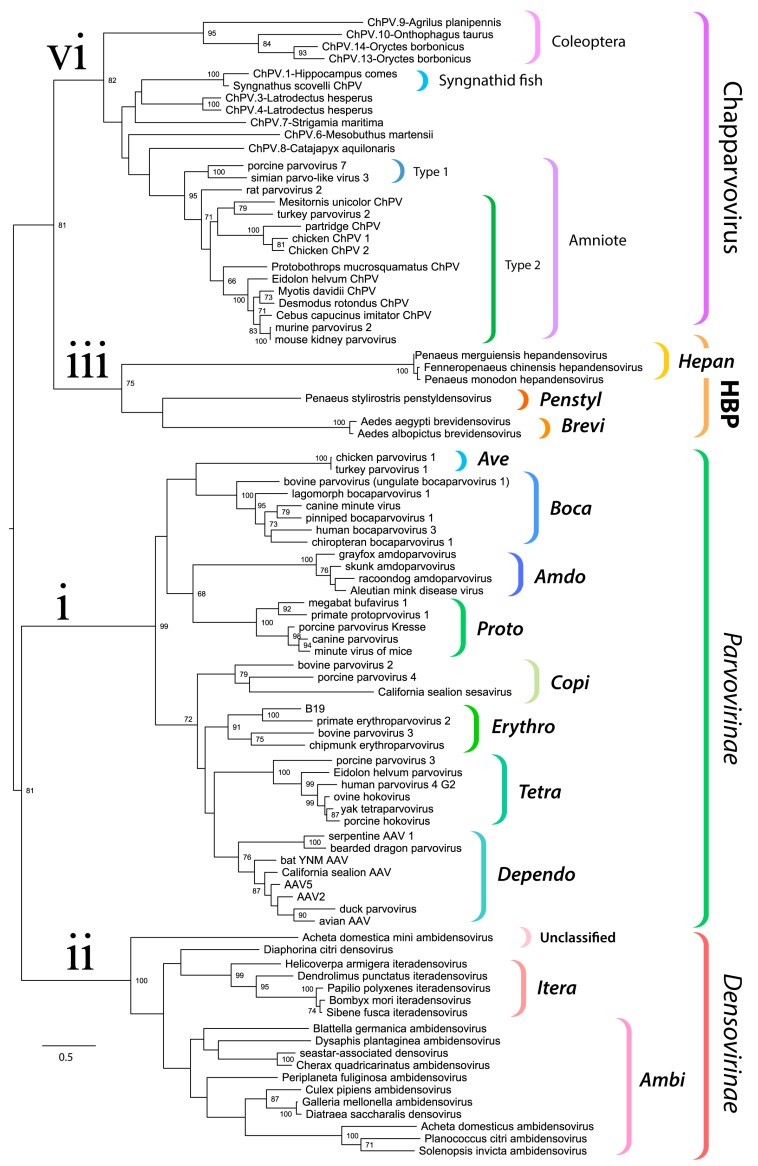
Evolutionary relationships within the family *Parvoviridae* reconstructed via phylogenetic analysis of the tripartite helicase domain. The four major splits within the *Parvoviridae* are indicated in the tree as follows: (i) *Parvovirinae* (ii) *Densovirinae* (excluding genera *He­pan*-, *Brevi*-, and *Penstyldensovirus*, abbreviated as HBP); (iii) HBP; (iv) Chapparvovirus (ChPV-related viruses and EPVs). Brackets to the right indicate taxonomic groups. The names of established genera are shown in bold italics in the abbreviated form (i.e., with the suffix “parvovirus” omitted). The scale bar shows evolutionary distance in substitutions per site. Numbers adjacent to tree nodes show bootstrap support (based on 100 bootstrap replicates) where >70%.

We used maximum likelihood-based phylogenetic approaches and an alignment spanning the tripartite helicase domain of the NS protein to reconstruct the evolutionary relationships of ChPVs, ChPV-derived EPVs, and previously reported parvoviruses (Figure 3). Strikingly, reconstructions indicated that the family *Parvoviridae* consists of four major clades, rather than the two that have historically been recognized [1]. Of these four lineages, one corresponds to the subfamily *Parvovirinae* as in current taxonomic schemes. However, the subfamily *Densovirinae* is split into two clades; one encompassing all ambisense densoviruses along with viruses of the genus *Iteradensovirus* (which have monosense genomes) and the second, referred to here as HBP, containing the *Hepan*-, *Brevi*-, and *Penstyldensovirus* genera. Moreover, a fourth parvovirus lineage was evident, comprised of the ChPVs and ChPV-derived EPVs.

**Figure 4:**
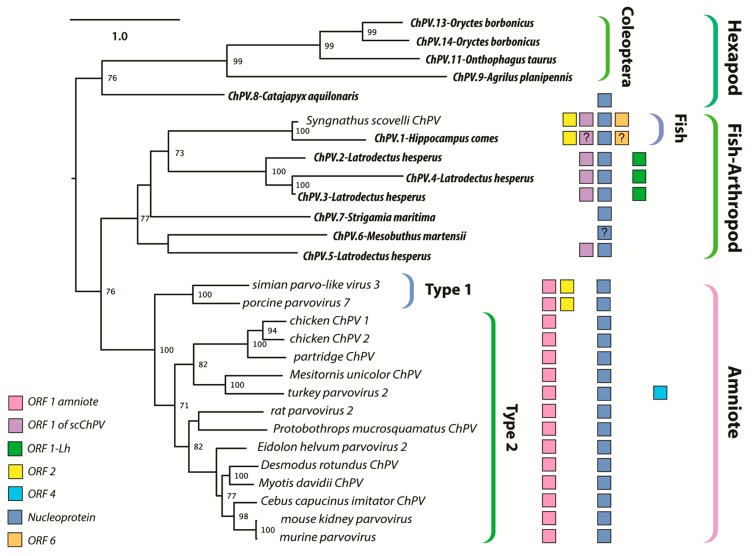
Maximum likelihood phylogenetic reconstructions of the ChPV clade based on the complete aligned amino acid sequences of the NS1. Colored boxes indicate the presence of auxiliary open reading frames (ORFs), as shown in the inset key. The “?” character indicates that the presence of an ORF is suspected but not confirmed. Taxa labels in bold italics indicate endogenous sequences, whereas italics indicate sequences known or believed to derive from viruses. Brackets to the right indicate taxonomic groups. The scale bar (top right) shows evolutionary distance in substitutions per site. Numbers adjacent to tree nodes show bootstrap support (based on 100 bootstrap replicates) where >70%.

The branching relationships between ChPVs were not fully resolved by phylogenetic analysis of the helicase domain. The putative large non-structural proteins (NS1) of ChPVs displayed a high degree of amino acid variability, particularly toward their N- and C-term. However, a region ~500-aa-long could be aligned reliably throughout all complete and partial entries previously proven to cluster within the ChPV lineage in the case of the NS helicase-based inference. Phylogenies reconstructed from this alignment reveal the ChPV-related viruses to be comprised of three robustly supported monophyletic lineages (Figure 4). One of these includes ChPVs sampled from amniotes (reptiles, birds, and mammals), in which two robustly supported sublineages (labeled type 1 and 2) were observed, corroborating the helicase-based phylogeny. The amniote ChPVs form a sister clade to EPVs found in the arthropod subphyla Chelicerata (arachnids, camel spiders, scorpions, whip scorpions, harvestmen, horseshoe crabs, and kin) and Myriapoda (millipedes, centipedes, and kin) as well as syngnathe fish. A third lineage was also observed, containing sequences from the arthropod subphylum Hexapoda (insects, springtail, and forcepstail). Within this lineage, the beetle EPVs formed a well-supported monophyletic clade.

### 3.3. Characterization of Syngnathid ChPVs and EPVs

**Figure 5:**
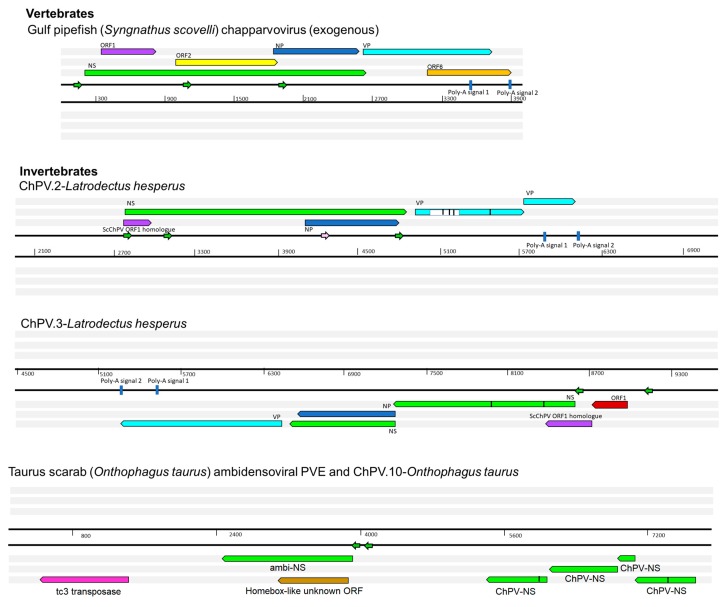
Genomic structures of newly identified chapparvoviruses (ChPVs) and ChPV-derived endogenous parvoviral element (EPV) sequences. The positions of putative open reading frames (ORFs) and predicted cis transcription elements of ChPVs are shown. ChPV.2-LatHes contains a previously unidentified repetitive element, present as multiple copies scattered in the *Latrodectus* genome, marked by the white box within the VP gene. The element ChPV.10-OntTau shares its integration site with another endogenous element, disclosing similarity to ambidensoviruses. ORFs are represented by rectangular arrows, colored according to homology. In-frame stop codons are shown as vertical lines. Splice donor sites are marked by white-colored bars, acceptor sites by orange-colored bars. Blue-colored bars show predicted polyadenylation signals. Small arrows show predicted promoters and are colored according to prediction score (>0.95 = green; 0.9–0.95 = pink: <0.9 = yellow). Grey boxes indicate regions inferred to be transcribed but not translated.

The ScChPV genome encodes a long NS ORF (807 aa), a strikingly short VP (367 aa), and a ChPV-like NP (Figure 5). Furthermore, a homologue of the ORF2 protein found in the amniote parvoviruses PPV7 and SPV3 was present. A predicted ORF was present in a genomic position equivalent to that of ORF1, found in amniote ChPVs. However, the predicted protein sequence did not disclose any detectable similarity to its amniote counterpart. ORF6, identified in partial overlap with the VP C-term encoding region, encodes a small protein of 27.2 kDa (239 aa), exhibiting no detectable similarity to any other sequence in GenBank. Fold recognition, however, revealed a potential structural similarity to viral structural proteins, including the major envelope glycoprotein of the Epstein–Barr virus (PDB ID: 2H6O chain A, *p* = 0.012), the minor viral protein of the Sputnik virophage (PDB ID: 3J26, chain N, *p* = 0.017) and the surface region of *Galleria mellonella* ambidensovirus (PDB ID: 1DNV, *p* = 0.021). These findings imply ORF6 may encode an auxiliary structural protein.

The partial ChPV-like sequence identified in the genome of the tiger seahorse (*Hippocampus comes*) was flanked by extensive stretches of host genomic sequence, establishing that, unlike the ScChPV sequence identified in the Gulf pipefish genome assembly, it likely represents an EPV rather than a virus. Interestingly, however, phylogenies showed that both sequences obtained from syngnathe fish are relatively closely related, and cluster together with high bootstrap support (Figure 3 and Figure 4).

### 3.4. Characterization of ChPV-Derived EPVs in Invertebrate Genomes

Among the ChPV-derived EPVs we identified in invertebrates, the most complete were identified in the western black widow spider (*Latrodectus hesperus*) (Figure 5). Two of these elements spanned near complete genomes, including *rep*, *cap,* and NP genes, and a homologue of the ORF1 gene found in ScChPV. In addition, the ChPV.2-LatHes element encodes an apparently complete NS protein (690 aa), while ChPV.3-LatHes discloses an undisrupted ORF1 (113 aa) as well as an apparently intact *cap* gene encoding a 386-aa-long VP. The putative NS1 proteins encoded by these elements displayed only 62% identity at aa level. The disrupted *cap* gene of ChPV.2-LatHes was found to include an insertion of 74 aa, suspected to originate from a yet unknown repetitive element (revealed by sequence comparisons to be interspersed throughout the *L. hesperus* genome).

ChPV.3-LatHes, on the other hand, appeared to include an intact upstream region of the genome, revealing an additional small ORF of 81 aa length directly upstream of the ScChPV ORF1 homologue, designated ORF1-Lh. This ORF disclosed no detectable homology to any sequences to date. Upstream of this ORF, a potential promoter sequence could be identified with high confidence (0.98 of 1). Both elements included complete, NP-encoding ORFs of 233 aa, although a canonical ATG start codon could only be identified in one element (ChPV.3-LatHes).

We identified two further elements in the western black widow spider genome, although these only spanned disrupted *rep* genes. ChPV.4-LatHes encodes nearly complete NS and NP genes, as well as a complete homologue of the ScChPV ORF1 gene. The true extent of preservation could not be assessed for this EPV as it occurs on a short scaffold that terminated within the EPV *rep* sequence. The putative NS1 of ChPV.4-LatHes was 80% identical to its counterpart in ChPV.2-LatHes at aa level. Interestingly, this element contains additional, *rep-*encoding regions directly upstream of a larger, NS1- and ScChPV ORF1-encoding region. This second region encodes only the first 221 aa of the putative NS1 protein, together with the putative ScChPV ORF1 homologue and ORF1-Lh genes. The ORF1-Lh gene encoded by ChPV.4-LatHes lacks an ATG start codon. The upstream promoter was weakly predicted, with a score of 0.6. ChPV.5-LatHes displayed a highly divergent, partial *rep* of 216 aa, with only 42% identity to the ChPV.2-LatHes NS1 at aa level (Figure 5). This element clustered outside the monophyletic clade defined by the three other *Latrodectus* EPVs (Figure 4).

A single ChPV-derived EPV was identified in a second arachnid species—the Chinese golden scorpion (*Mesobuthus martensii*). This element was identified in a relatively short, unplaced scaffold, and comparison to the *Latrodectus* elements indicated that the contig was truncated within the EPV sequence, consequently the true extent of its preservation could not be assessed. Nevertheless, ORFs disclosing homology to the NS, NP, and VP proteins could be identified (Figure 2). While the first 100 or so codons of the NS ORF were absent, a complete NP ORF was detected, along with the first 46 codons of VP. All three ORFs were disrupted by frameshifts and stop codons. No homologues of any alternative ORFs identified in other ChPV genomes could be identified.

We identified a ChPV-derived EPV in the genome of a myriapod—the European centipede (*Strigamia maritima*). This element displayed partial homologues of the NS and NP encoding ORFs, both of which contained large deletions (Figure 2) as well as numerous nonsense mutations (Table 1). Moreover, the NS ORF was disrupted by an extensive stretch of an insertion of unknown origin. No homologues of any of the alternative ORFs found in other ChPVs could be identified in this endogenous sequence.

Seven ChPV-derived EPVs were identified in hexapod arthropods (subphylum Hexapoda). One occurs in the genome of a bristletail species—the Northern forcepstail (*Catajapyx aquillonaris*)—belonging to the entognath order Diplura. The other six were identified in three species belonging to the vast insect order Coleoptera: the emerald ash borer (*Agrilus planipennis*), the taurus scarab (*Onthophagus taurus*), and the scarab beetle (*Oryctes borbonicus*). The bristletail element contains a C-terminal truncated *rep* of at least 250 aa and a near full-length NP ORF. The partial *rep* was intact, but the NP ORF is disrupted and highly divergent, showing significant sequence similarity only in the conserved core region of the putative protein. The ash borer element ChPV.9-AgrPla occurs in a scaffold that is ~1 kb in length. One end of this scaffold contains a 592 nt region exhibiting homology to the NS ORF, which harboured an N-terminal deletion of at least 200 aa. 

In ChPV.10-OntTau, a disrupted but almost complete NS ORF could be identified (Figure 5). Interestingly, a second EPV insertion was detected at the same locus. This element encodes an intact, potentially fully-expressible NS gene, homologous to the NS1 of ambidensoviruses (genus *Ambidensovirus*) and disclosing similarity to a recently reported ambidensovirus sequence that has been detected only at cDNA level in the transcriptome of two bumble bee species (*Bombus cryptarum* and *B. terrestris*) [40]. An additional intact, potentially expressible ORF was present in this ambidensoviral element, overlapping the putative NS1 gene, which harboured no significant similarity to any sequences deposited in GenBank to date. In its derived aa sequence, however, a homeobox domain could be revealed. The other two elements of the taurus scarab genome were located together in another assembly scaffold, only 2540 nts apart from each other. Both EPVs consisted of only a partial ORF, which disclosed similarity to chapparvoviral *rep*s. None of these elements encompassed the tripartite helicase domain, hence they were not included in the phylogenetic inference.

Two EPVs were identified in the scarab beetle genome. One of these, designated ChPV.13-OryBor, harboured a near complete *rep* at 402 aa, as well as a short, partial *cap*, capable of encoding only the first 33 aa of the putative VP. The region of *rep* homology occurred within an ORF that was not disrupted by any frameshifts and could be extended without disruption upstream and downstream, suggesting that a longer gene product—potentially encoding a longer, divergent NS protein—may be present. However, these regions did not disclose sequence similarity to any proteins hitherto deposited to GenBank. The ChPV.14-OryBor element included only a heavily truncated NS of 254 aa.

### 3.5. Structural Characteristics of ChPV Capsids

**Figure 6:**
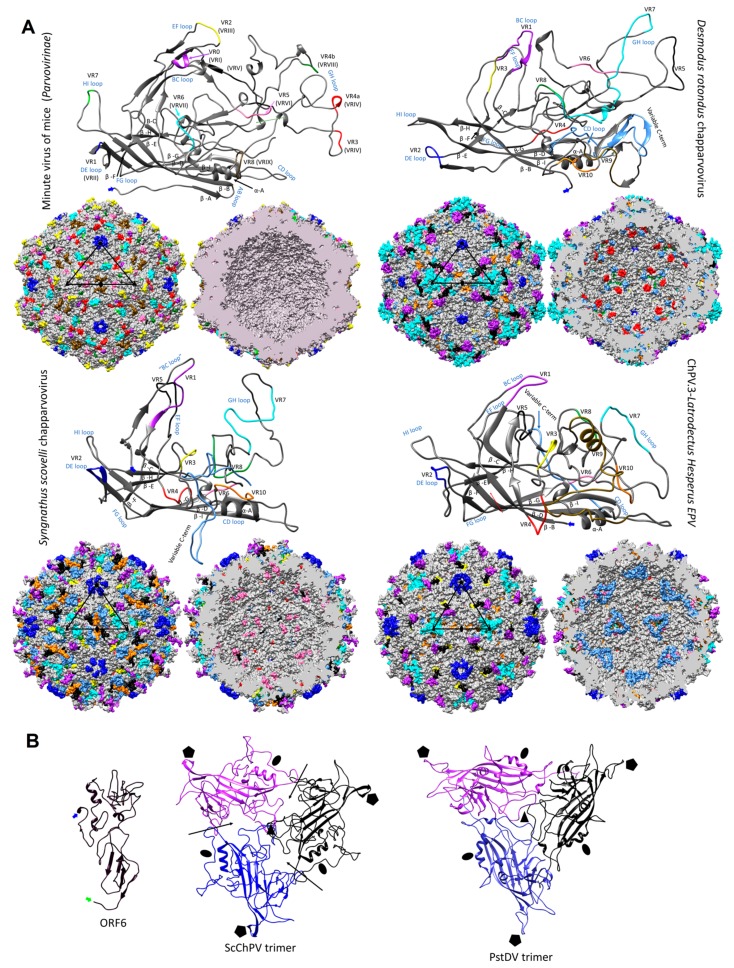
Structural variation and assembly interfaces of chapparvoviruses (ChPVs). (**A**) Comparison of VP monomer ribbon diagrams of the protoparvovirus minute virus of mice (PDB ID: 1Z14) from subfamily *Parvovirinae* to homology models of an amniote, a fish, and a ChPV-derived EPV from an arthropod genome (ChPV.3-LatHes). Variable regions (VRs) of the same number are marked by the same color and mapped to the surface and luminal area of the *T* = 1 icosahedral capsid model constructed of 60 monomers. In the case of the minute virus of mice, the VRs are marked by both the traditional numbering established for dependoparvoviruses (Roman numerals) and by the special numbering applied for protoparvoviruses only (Arabic numerals). Blue signs indicate the names of the loops linking the beta strands of the conserved jelly roll core. Triangles mark the position of an asymmetric unit within the capsid, the five-fold symmetry axis is marked by a pentagon, the three-fold with the black filled triangles, and the two-fold with an ellipsoid. (**B**) Homology model of ORF6, the hypothetical structural protein of *Syngnathus scovelli* ChPV (ScChPV). The trimer of the ScChPV monomer model reveals a gap at each subunit interaction (arrows), unlike in the case of the trimer of even the hitherto smallest parvoviral capsid protein, *Penaeus stylirostris* densovirus. The gap might accommodate ORF6 in the assembled ScChPV capsid. Symmetry axes are marked by the same symbols as for panel A.

We built 3D homology models to facilitate the comparison of ChPV capsid structures to those found in other parvoviruses. Interestingly, structural similarity with erythro-, proto-, and bocaparvoviruses can be detected for VP using fold recognition, even though the VP proteins of ChPVs share no significant sequence similarity with those of other parvoviruses (Figure 6a).

The derived polypeptide sequence of the complete VP ORF encoded by DrChPV was subjected to fold recognition, to identify suitable templates for homology modeling. This comparative analysis showed that the VP2 protein of parvovirus H1 (genus *Protoparvovirus*) (PDB ID: 4G0R) could potentially harbor the most structural similarity (*p* = 9 × 10^−5^), and this sequence was therefore used as the template for homology modeling. Due to the lack of sequence identity and the non-homologous nature of the ChPV VP genes to other parvoviral VPs, we used the final model obtained in this analysis as a template to construct homology models for four further ChPV VPs—rat parvovirus 2, PPV7, TPV2, and pit viper ChPV. This allowed us to overcome the stochastic aspect of model construction. Although the pitfalls of using models as templates have to be noted, this approach ensured that only those regions showed structural variability which would likely do so in the actual capsid structures.

We examined the VP sequences of two representatives of the second major ChPV clade (see Figure 4)—one derived from a presumably exogenous virus (ScChPV) and one from an EPV (ChPV.3-LatHes). For the VP protein encoded by ScChPV, fold recognition identified the following dependoparvovirus VP3 proteins as potential templates: adeno-associated virus 8, PDB ID: 2QA0, *p* = 9 × 10^−4^; Adeno-associated virus rh32.33, PDB ID: 4IOV, *p* = 9 × 10^−4^, while for the VP encoded by the black widow spider EPV the most reliable hit was the VP4 protein of an iteradensovirus (*Bombyx mori* densovirus 1, PDB ID: 3P0S, *p* = 8 × 10^−4^). When superimposing the obtained models with the VPs of AAV8 and BmDV1, however, structural similarity only covered the jelly roll core and the αA helix, and of the surface loops traditionally considered more variable, only the BC loop.

Modeling indicated that the ChPV VP monomer harbors an eight-stranded β-barrel “jelly roll” core and the αA helix at the two-fold symmetry axis, as found in all members of the family *Parvoviridae* to date [41] (Figure 6a). Equivalents of all short strands were present (β-C, H, E, F) as well, for four out of the five longer strands (β-B, D, I, G). However, no structural analogue to the outmost β-A could be identified (Figure 6). Examining the secondary structure prediction confirmed that a β-A analogue was not present, indicating β-B to be the closest to the N-term. The first strand of the *Syngnathus scovelli* ChPV VP appeared to fold outside of the jelly roll, leaving the longer sheet of the barrel without a β-B, comprised of only three strands—namely D, I, and G—despite a complete upper, CHEF sheet (Figure 6a). When modeling the complete *T* = 1 capsid polymer, this manifested as a hole, which is normally covered by β-B, even in the case of the smallest parvoviral capsids (Figure 6b). All VPs encoded by ChPVs and ChPV-derived EPVs displayed two canonical loops surrounding their five-fold axes, linking sheets D with E at the five-fold channel and sheets H with I on the floor surrounding the channel. In case of the amniote ChPVs, the pore displayed a tight opening. The sequence of the DE loop varied to some extent among these seven sequences, which also manifested in the models. The HI loop was, however, highly conserved throughout, containing only one variable position between the amniote ChPVs.

We mapped the chapparvoviral VRs identified by VP alignments (Figure S3) to both VP monomers and complete capsids, to examine how they manifest on the virion surface and make comparisons to parvoviruses of known structure, represented by the minute virus of mice (MVM), the prototypic member of subfamily *Parvovirinae* (PDB ID: 1Z14) (Figure 5). Out of ten chapparvoviral VRs identified (VR 1 to 10), shown in Figure S3a, only VR1, VR2, and VR9 proved to be similarly positioned and hence likely analogous to their counterparts in the MVM capsid. Some VRs (VR4 in all ChPVs examined, VR8 of the amniote ChPVs, and VR6 in ChPV.3-LatHes and ScChPV) appeared to be positioned at the luminal surface of the ChPV capsid, distinct from all parvoviruses studied to date. The only exception, however, is bovine parvovirus, a bocaparvovirus [42] in which VR8 is also located on the luminal surface of the capsid. Since the ChPV VRs appeared to be non-homologous to those established for either proto- or dependoparvoviruses, we re-defined them by numbering from N to C-term.

In addition to their distinctive VRs, ChPVs ubiquitously appeared to harbor a highly variable C-terminal region, with a length varying between 12 and 62 residues. The ChPV VP variable C-term appears to be buried in most cases, with the exception of ScChPV, where it is probably exposed. In the VP encoded by ChPV.3-LatHes it forms the luminal surface of the three-fold, whereas in the case of fish and amniote ChPVs it is located at the two-fold (Figure 6a).

The ScChPV and ChPV.3-LatHes VP lacked a VR6 homologous to that of the amniote ChPVs, albeit displayed variation in another position instead, still in the sixth-place counting from the N-term (Figure S3b). Moreover, both of them displayed truncated VRs 3, 5, and 7, compared to their amniote counterparts. VR9, furthermore, was absent from the ScChPV VP, whereas VR10 was missing from the VP of ChPV.2-LatHes (Figure S3b). As for the surface, the largest variable region for amniote ChPVs, namely DrChPV, is VR7, forming the entire three-fold protrusions, with VRs 1, and 9 forming small protrusions surrounding the aforementioned peaks.

**Figure 7:**
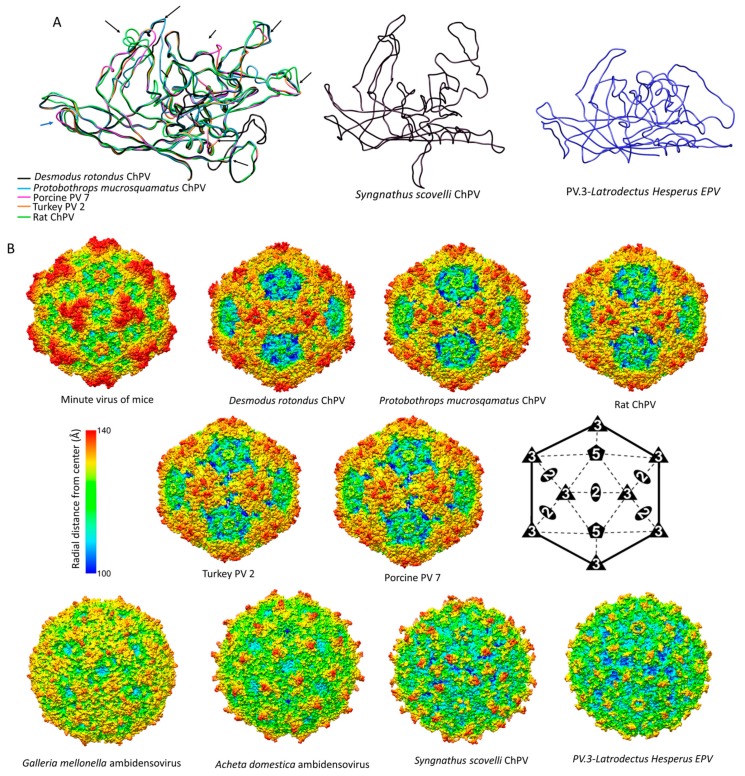
Comparison of chapparvoviruses (ChPV) capsid models of various host affiliations. (**A**) Homology models, shown as ribbon diagrams, representing the probable three different ChPV structural protein types. The first panel shows superposition of VP monomer homology models of amniote ChPV capsids, including reptilian, avian, rodent, chiropteran, and ungulate representatives. Black arrows show variable regions (VRs) previously identified by aligning the VP protein sequences. The next two panels show homology models of capsid monomers from a fish ChPV and an endogenous chapparvoviral element from an arthropod genome. (**B**) Capsid surface morphology of amniote ChPV homology models compared to that of the polymer structure of a prototypic parvovirus, the minute virus of mice (MVM) (PDB ID: 1Z14 at 3.25 Å resolution). Capsids are orientated by their two-fold symmetry axes, as shown in the line diagram, and are radially colored. Below, the comparison of homology models of complete viral capsid surface morphology of the newly identified fish ChPV and arachnid endogenous chapparvoviral element is shown, with that of the actual capsid structure of two densoviruses (subfamily *Densovirinae*, genus *Ambidensovirus*) (PDB ID: 4MGU at 3.5 Å resolution for *Acheta domestica* densovirus and 1DNV at 3.7 Å for *Galleria* densovirus).

The complete capsid models of non-amniote ChPVs were observed to harbor surface features that are strikingly distinct from those of the amniote ones, more closely resembling the capsids of the *Ambidensovirus*-*Iteradensovirus* clade of *Densovirinae* (see Figure 3), with a surface that is less spikey (Figure 7a). The homology model of ORF6 of ScCHPV, constructed based on the minor viral protein of the Sputnik virophage (PDB ID: 3J26) indicates that this potentially structural protein harbors multiple beta strands close to its C-term, out of which the outermost could potentially fill in the aforementioned gap caused by the lack of a β-B (Figure 6b).

## 4. Discussion

Historically, the family *Parvoviridae* has always been comprised of two subfamilies, with specificity for vertebrate or invertebrate hosts being the major demarcation criterion [2]. This division was initially supported by phylogenetic inference. However, as the number of densoviral genera increased, the heterogeneity of densoviruses, specifically their segregation into two clades, has not gone unnoticed [1]. Our study provides further evidence that the traditional division of parvoviruses into vertebrate-specific and invertebrate-specific subfamilies no longer holds, rather, it supports the division of the *Parvoviridae* into four major subgroups: the *Parvovirinae*, a split *Densovirinae*, and the ChPVs, as illustrated in Figure 3.

The data presented here show that ChPVs infect an exceptionally broad range of hosts, including both vertebrates and invertebrates. We show that ChPVs found in fish are more closely related to those that infected ancestral arachnoid arthropods than they are to those that infect amniote vertebrates (Figure 4), suggesting that ChPVs may have been transmitted between distantly related host species in the past. Furthermore, phylogenies indicate that all amniote ChPVs have a common origin (Figure 3), consistent with the overall conservation of their genome organization and some aspects of predicted transcriptional strategy (Figure 1).

While previous studies have suggested that ChPVs broadly co-diverged with host species [9], the present, expanded data set reveals that some transmission of ChPVs between vertebrate classes may have occurred (Figure 4). However, it should be kept in mind that almost all amniote ChPVs have been identified via metagenomic sequencing of environmental samples (mostly fecal viromes) and their true host affiliations remain uncertain.

The EPV sequences found in animal genomes overwhelmingly derive from a small proportion of parvovirus lineages [13,14,17,43,44]. For example, ambidensovirus-derived EPVs dominate invertebrate genomes [14], whereas vertebrate EPVs almost exclusively derive from the *Dependoparvovirus* and *Protoparvovirus* genera [12,13,43,44]. In this study, we found no trace of ChPV-derived EPVs in amniote genomes, despite recent evidence that ChPVs infect this host group [21,45]. By contrast, ChPV-derived EPVs are relatively common in arthropods, with some species harboring multiple, independently acquired elements, occasionally even in close proximity within the host genome (Table 1, Figure 4). The tendency of EPVs to derive from a subset of parvovirus genera likely has biological underpinnings. For example, in vertebrates it may reflect the ability of dependoparvoviruses to integrate into host DNA, and/or the requirement of protoparvoviruses to initiate DNA damage response (DDR) during replication [46,47]. Similar features of the viral life cycle could account for the biased distribution of ChPV-related sequences in animal genomes, i.e., arthropod and fish ChPVs might have adopted a replication strategy that favors germline integration, whereas that of amniote ChPVs precludes it. Notably, some arthropod species have integration sites containing multiple independently acquired EPVs of both ChPV and ambidensovirus origin, suggesting that hotspots of parvovirus integration and/or fixation might exist in their genomes.

Our discovery of ChPV-derived elements in fish and arthropod genomes establishes that ChPVs can infect these species in addition to amniotes [21,45]. Moreover, it provides evidence that the ChPVs are likely an ancient lineage of parvoviruses. Though we did not identify any orthologous ChPV insertions, the EPVs described here show extensive evidence of germline degradation. Through comparison to studies of EPVs in mammals (in which several orthologous EPVs have been described [13,48]), it appears likely that ChPVs have been present in animals for many millions of years. Moreover, as the hexapod EPVs appear to be monophyletic and mirror the evolution of their host species, the age of ChPVs could possibly correlate with the Insecta–Entognatha split, suggesting a minimum age of 400 million years [49].

Through comparative analysis of EPVs and ChPVs, we show that ChPV genomes exhibit a number of defining characteristics. Firstly, all possess a short, monosense genome, encoding a relatively large NS and a relatively short VP. The short VP proteins of ChPVs are clearly homologous to one another, but show no similarity to those found in other parvovirus lineages. Similar to those found in the penstyl-, hepan-, and brevidensoviruses, the VP proteins of ChPVs lack the phospholipase A2 (PLA2) domains that are required for infectivity in most other parvoviruses. Notably, these are also the genera to which ChPVs are most closely related in NS-based phylogenies (Figure 3). 

Secondly, ChPVs typically encode multiple additional gene products besides the NS and VP. To begin with, almost all encode a nucleoprotein (NP) gene in an overlapping frame with *rep*. In this report, we show that putative NP ORFs are present in ChPV-derived EVEs, suggesting it is an ancestral, conserved feature of these viruses. However, its absence from the coleopteran lineage is intriguing, as it is still present in the EPV of the hexapod stem group Diplura of Entognatha. Phylogenetic reconstructions (and the extensive overlap with *rep*) imply it was acquired ancestrally and independently lost in the lineage derived from members of the hexapod crown group, Coleoptera (Figure 4).

A functional role for auxiliary ORF1 is supported by: (i) its conservation across the entire amniote ChPV clade; and (ii) limited experimental data indicating it is expressed in MKPV via a spliced transcript. Auxiliary ORF2 was only identified in a small subset of ChPV genomes, but a functional role for this ORF is suggested by the presence of homologues in distantly related ChPVs of amniotes and fish (see Figure 4). Interestingly, although all ChPVs appear to express ORF1 via splicing of a small intronic sequence (Figure 1), those harboring an ORF2 homologue are predicted to lack the peculiar large introns found in the expression of MKPV NP and VP transcripts [21]. ScChPV lacks an ORF1 homologue, but contains a predicted reading frame in the corresponding position. Homologues of this ScChPV ORF1 variant are present in all three arachnid EPVs, although not in the first, but in the second position. As only the three *Latrodectus* EPVs possess a homologue of ORF1-Lh, it is possible that this small ORF originated after the split from the syngnathe fish lineage, whereas the ScChPV ORF1 originates earlier. The distribution of homologous auxiliary genes across phylogenetic lineages of ChPVs implies that distinct lineages have acquired and/or lost these genes on multiple, independent occasions. 

MKPV has been reported to possess only one promoter and two polyadenylation signals, as well as an extensive number of spliced transcripts. This transcription pattern, however, appears to be unique to only one of the two hitherto amniote ChPV lineages, comprising of rodent, chiropteran, New World primate, avian, and reptilian entries. As members of the “type 1” lineage, including PPV7, appear to display a genome organization specific for this clade and different from that of MKPV, they may utilize distinct transcription strategies as well. 

Despite the potential pitfalls of homology modeling, and the use of distinct templates to reconstruct both the VP monomer and capsid structures, we obtained remarkably similar predicted structures for VP sequences found in closely related viruses/EPVs. Since the viral capsid plays an important role in mediating the interactions between parvoviruses and their hosts, comparisons of capsid structures can potentially reveal insights into parvovirus biology. Our analysis indicates that ChPV VPs would assemble into a complete, *T* = 1 icosahedral capsid, despite their relatively small size. Furthermore, their predicted structures are remarkably similar to those found in other parvoviruses, despite the lack of any detectable similarity in the sequences of their VP proteins. Structural similarities include the presence of a conserved jelly roll core and α-A helix, the existence of the D–E and H–I loops, and the presence of identifiable VRs. Interestingly, the amniote ChPV capsids appear to possess the same number of VRs as most of the vertebrate parvoviruses of subfamily *Parvovirinae*, even if only a few of them (namely VRs 1, 2, and 9) proved to be analogous features. In these virus capsids, variations were most prominent among the three-fold peaks and protrusions, as well as the two-fold depression, as observed in members of the *Parvovirinae* (Figure 7). The tendency of some VRs to manifest at the luminal surface of the capsid in models suggests these regions could play a role in intracellular host–virus interactions. For these regions to become accessible to intracellular signaling pathways would require either uncoating or conformational changes. Based on previous findings, however, the parvovirus capsid appears to traffic into the nucleus intact [50,51]. Considering this, these buried regions might play a role in processes linked to the nucleus. Interestingly, bovine parvovirus, the only other parvovirus in which buried VRs have previously been observed [52] is an enteric pathogen, and the association of amniote ChPVs with fecal viromes suggests these viruses might also be largely enteric. 

In addition to the VRs, all ChPVs seem to harbor highly variable VP C-terms. A similar phenomenon has been observed in the case of iteradensoviruses, in which the last 40 C-terminal residues are disordered, hence the structure of this region cannot be resolved [53]. Although the location of the ChPV C-term appears to vary, its association with regions that are overtly involved in parvovirus–host interactions (e.g., the two- and three-fold peaks) is certainly intriguing. 

MKPV is associated with the pathology of the urogenital system, whereas a related virus, murine ChPV, has been detected at a very high prevalence in murine liver tissue, suggesting it is a gastrointestinal agent [45]. The VPs of the two, however, only differ in six aa residues, located within VR3 and near VR2 on the surface and in the buried VR4, as well as in the similarly buried variable C-term (Figure S3a). Thus, these positions could constitute potential determinants of tissue tropism in murine ChPVs. 

Parvovirus subfamilies *Parvovirinae* and *Densovirinae* utilize distinct strategies to stabilize their icosahedral capsids [54]. Vertebrate parvoviruses extend the longer side of the jellyroll fold with an additional, N-terminal strand by folding back β-A to interact with the two-fold axis of the very same monomer, hence creating an extended ABDIG sheet [55,56]. By contrast, the densovirus capsid preserves the symmetric arrangement of the jellyroll fold, and possesses a β-A which is a direct elongated N-terminal extension of the β-B instead, interacting with the β-B strand of the neighboring monomer toward the five-fold axis [57,58]. Strikingly, our data show that ChPV capsids lack β-A strands (and also the β-B strand, in the case of ScChPV). The functional implications of this are unclear—possibly ChPV capsids are stabilized in the absence of β-A via a yet unknown, additional VP. If ChPVs express additional structural proteins, they are presumably encoded by spliced transcripts (given the unusually small size of the *cap* gene). Alternatively, the ChPV capsid might assemble without the incorporation of an additional β strand, perhaps at the cost of losing the stability and resilience typical of parvoviruses in general. Potentially, this could account for the apparent presence of buried VRs. Interestingly, in studies of MKPV, viral proteins could be detected in the kidneys of infected mice, even though no assembled particles could be observed in inclusion body-affected tubular cells [21]. This, along with our structural predictions, suggests that the ChPV strategy for uncoating and cellular trafficking might be very different from that found in the *Parvovirinae* and *Densovirinae*.

Uniquely, the genome of ScChPV appears to include a putative additional structural protein (ORF6), in addition to the above-mentioned alternative ORFs. All parvoviruses to date—except those of genus *Penstyldensovirus,* with only one VP comprising the capsid [58]—have been reported to incorporate up to three additional minor VPs into the virion, which share a common C-terminal region. To encode a structural protein on an entirely separate ORF sharing, no mutual coding sequence with *cap* would be unique. Possibly, this unusual feature could be connected to the predicted lack of a β-B strand in the ScChPV VP monomer.

Taken together, the data presented here establish that the ChPVs belong to a parvovirus lineage that comprises a distinct lineage from all other parvoviruses, and infects an exceptionally broad range of host species, including both vertebrates and invertebrates. Consistent with this, their relatively complex genomes exhibit numerous unique features, implying that their life cycle might significantly differ from what has been established in the case of other members of the family. These findings underscore the need for further basic and comparative studies of ChPVs, to assess their potential impact on animal health, both wildlife and livestock. Furthermore, this is the first study to imply that vertebrate parvoviruses are not monophyletic, and that members of the family must have evolved to infect vertebrates on at least two separate occasions.

## Figures and Tables

**Table 1:** Novel ChPV sequences identified in this study.

Host Common Name	Host Scientific Name	Virus/Element Name ^a^	Gene Content	Nonsense Mutations ^b^
**Vertebrates**				
Gulf pipefish	*Syngnathus scovelli*	ScChPV	*rep+cap*	0; 0
Tiger tail seahorse	*Hippocampus comes*	ChPV.1-HipCom	*rep*	2; 2
**Invertebrates**				
Black widow spider	*Latrodectus hesperus*	ChPV.2-LatHes	*rep+cap*	4; 1
		ChPV.3-LatHes	*rep+cap*	3; 1
		ChPV.4-LatHes	*rep* *	3; 3
		ChPV.5-LatHes	*rep*	4; 2
Chinese scorpion	*Mesobuthus martensii*	ChPV.6-MesMar	rep+cap *	2; 3
European centipede	*Strigamia maritima*	ChPV.7-StrMar	*rep*	2; 3
Northern forcepstail	*Catajapyx aquilonaris*	ChPV.8-CatAqu	*rep*	0; 0
Emerald ash borer	*Agrilus planipennis*	ChPV.9-AgrPla	rep*	2; 0
Taurus scarab	*Onthophagus taurus*	ChPV.10-OntTau	*rep*	2; 3
		ChPV.11-OntTau	*rep*	0; 0
		ChPV.12-OntTau	*rep*	2; 1
Rhinocerous beetle	*Oryctes borbonicus*	ChPV.13-OryBor	*rep*	2; 0
		ChPV.14-OryBor	*rep*	0; 0

^a^ For sequences that are presumed to derive from viruses, the proposed name of the virus is shown. For endogenous parvoviral elements (EPV) the locus name is given, following the standard nomenclature proposed for endogenous retrovirus (ERV) loci [39], except using the classifier “EPV” in the place of “ERV”. The table shows a shortened version of the ID, used in the text of this manuscript, wherein the “EPV” classifier is omitted, and an abbreviated version of the species name is used within the taxonomic component of the ID (derived from the first three letters of each component of the Latin binomial scientific name of the host species). ^b^ Stop codons; frameshifts. * Asterisks indicate contigs that were truncated within the virus-derived portion of the sequence. Underlined names indicate the presence of the complete ORF.

## Supplementary Materials

The following are available online at https://www.mdpi.com/1999-4915/11/6/525/s1, Figure S1: Predictions of secondary structure, disordered regions and potential phosphorylation sites in case of an amniote exogenous and an endogenous invertebrate ChPV nucleoprotein (NP), Figure S2: Secondary structure predictions of the *Syngnathus scovelli* ChPV genome termini. Figure S3: Variable regions at the derived amino acid sequence level identified among ChPV capsid proteins.
