# Supplementary material for: An Ancient Lineage of Highly Divergent Parvoviruses Infects both Vertebrate and Invertebrate Hosts
Source: Viruses. 2019 Jun 6;11(6):525. doi: 10.3390/v11060525 (PMC6631224; doi:10.3390/v11060525)
Supplement: Supplementary file 1 [file viruses-11-00525-s001.pdf]

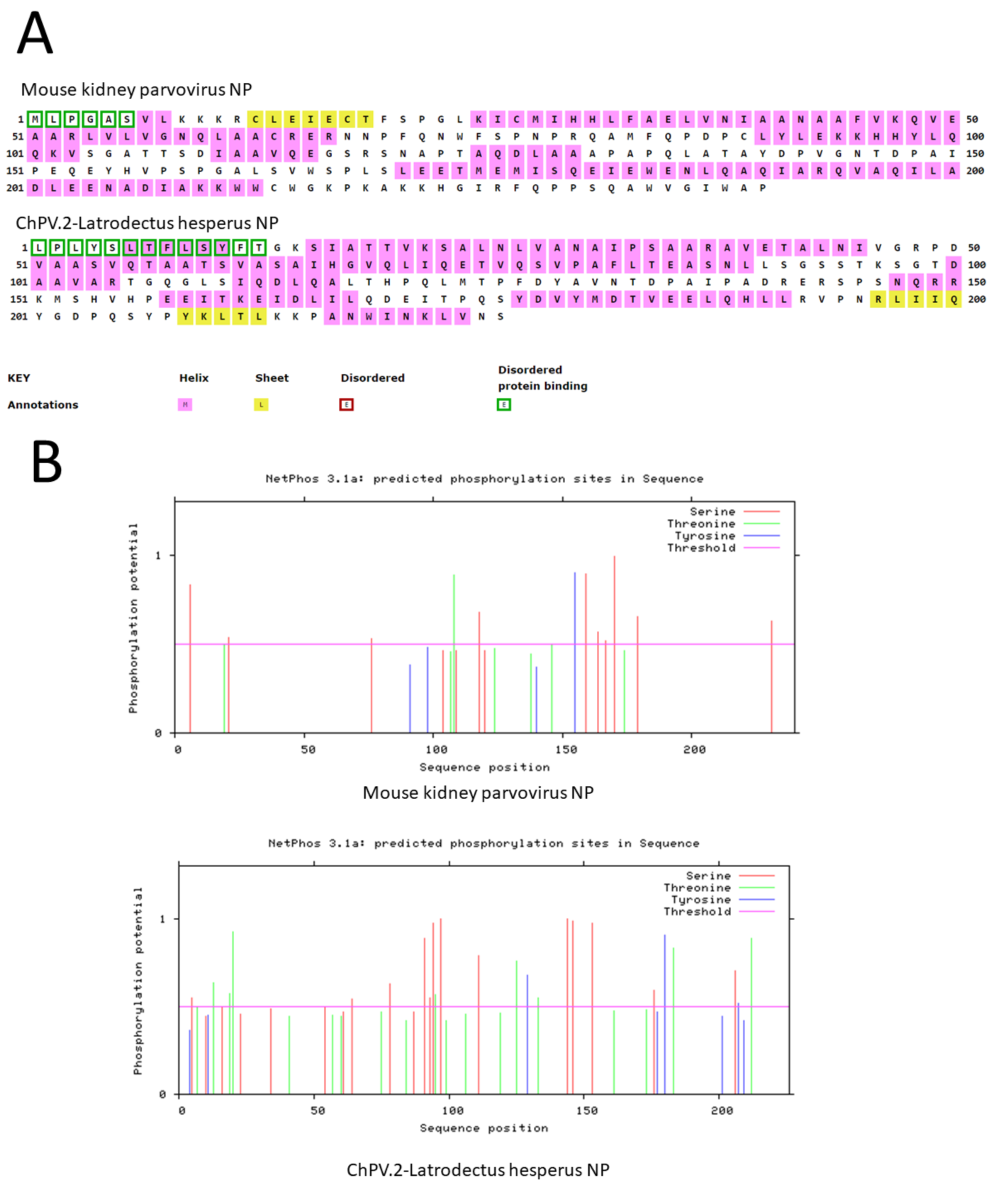
A
Mouse kidney parvovirus NP
ChPV.2-Latrodectus hesperus NP
KEY
Annotations
Helix
Sheet
Disordered
Disordered protein binding
B
NetPhos 3.1a: predicted phosphorylation sites in Sequence
Serine
Threonine
Tyrosine
Threshold
Phosphorylation potential
Sequence position
Mouse kidney parvovirus NP
NetPhos 3.1a: predicted phosphorylation sites in Sequence
Serine
Threonine
Tyrosine
Threshold
Phosphorylation potential
Sequence position
ChPV.2-Latrodectus hesperus NP

Figure S1

Predictions of secondary structure, disordered regions and potential phosphorylation sites in case of an amniote exogenous and an endogenous invertebrate chapparvovirus nucleoprotein (NP). (A) Secondary structure and disordered region predictions of NP from mouse kidney parvovirus and from the element ChPV.3-Latrodectus hesperus. (B) Predictions of potential phosphorylation sites in case of the same amniote chapparvovirus and an invertebrate endogenous chapparvoviral element NPs.

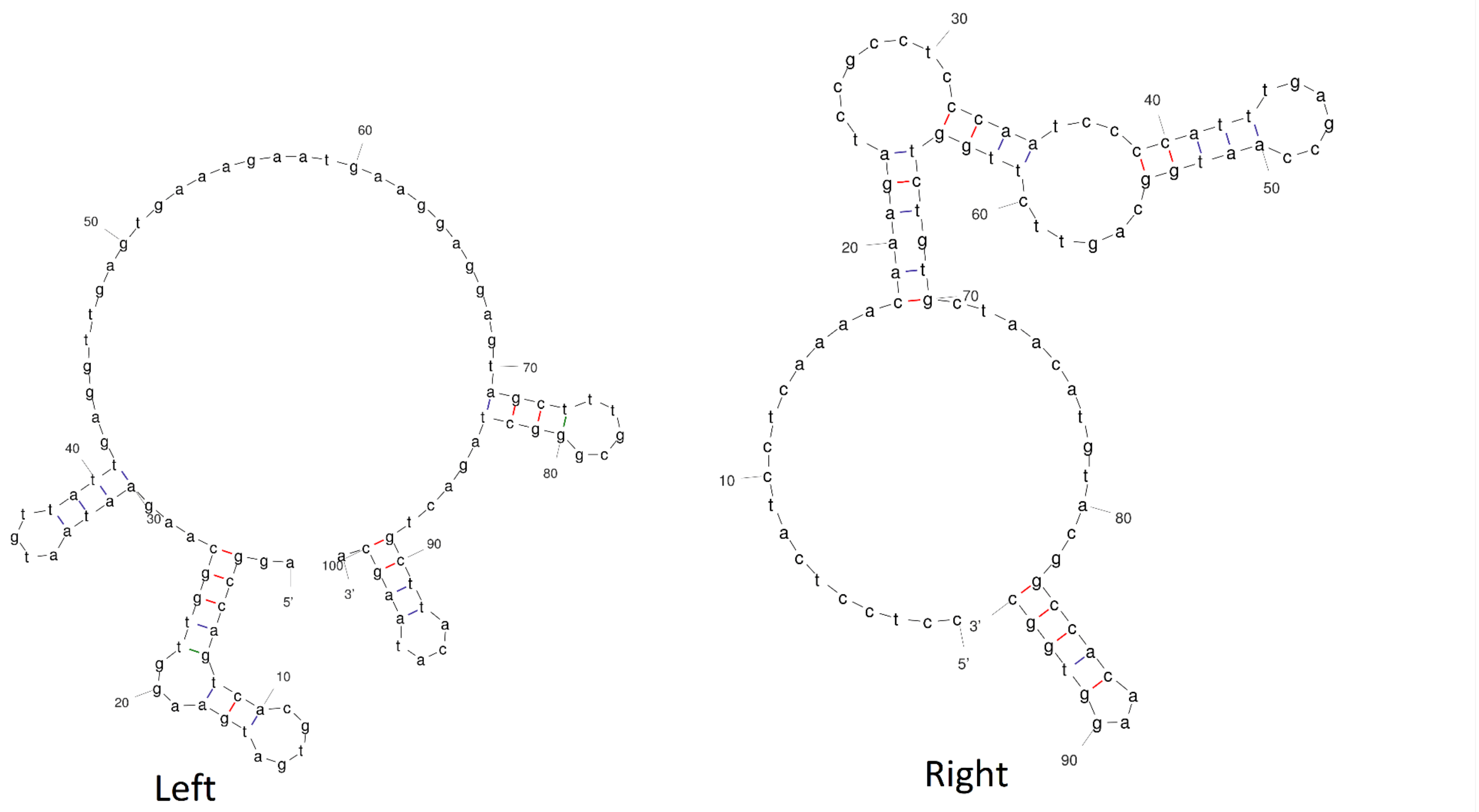

Figure S2

Secondary structure predictions of the *Syngnathus scovelli* chapparvovirus genome termini.

A

```
                                                VR1
murine_ChPV_NY     1 MAEDVTFHNTYMVYWKNQPFIYPNTNINPPN--AHTMSAGAINTGWHIIPTILWKHFLTPKQWTEFTINFEAYTVKGYSCTIYN  82
MKDV               1 MAEDVTFHNTYMVYWKNQPFIYPNTNINPPN--AHTMSAGAINTGWHIIPTILWKHFLTPKQWTEFTINYEAYTVKGYSCTIYN  82
rat2               1 MATDVSFSNVFMAYWSNSPYNYKVSGAGRDH--T-RPTGTPINTGWHILPLTLWKHFTTAKQWAELVINYEAYTVKGFSCTVYN  81
Desmodus_rotond    1 MAEDVSYTNTYMAYWKNGPYIYPNNDTDPKSKTGQVMSTGEINTGWHIIPTILWKHFTTPKQWCNFMIKYEAYTVKGYTVTVYN  84
porcine7           1 MAEHITLSNTFMAYWENDPYQYPTYKPFQKN------EILTSNTGWHILLNILWRHFLSPKQWYELCINYEAYHVEGTSTTVFN  78
turkey2            1 MADDISFSHSYMCYIKNQPYLYPTQKNPTMP------QESTLNTGWHIIPTTLWKHFCTPADWVDLITHYEAYSVTGYSVSIFN  78
pit_viper          1 MATDHVFTNTYMCYWENKPYLYPANQPGVKR------DGDVYTTGWHIIPNMLWRHVTTPKQWAEFQIQTEAYHVKSIKAIVYN  78

cons               1 ** .    : :* * .* *: *                    .*****:   **:*. :. :* ::  : *** * . .  ::*  84

                        VR2 – DE loop                            VR3                 VR4
murine_ChPV_NY    83 PIPMTQQLAIQGTTAFTAFNNTIYTLGAQDDLYETAYHNWYNDDATGDYKAFNLSFKEGQYK--------NF--NGSWKKTMWP 156
MKDV              83 PIPMTQQLAIQGTTAFTAFNNTIYTLGAQDDLYETAYHNWYSDDSTGDYKAFNLSFKEGQYK--------NL--SGSWKKTIWP 156
rat2              82 PVPMTQQLAIQGTTTFTAFNNTIYSLGVKDELYETSWYNWNDPAGTGGFNNFSLAYKEGYYL--------DSPRAATQKRTLLP 157
Desmodus_rotond   85 PIPMTQQLAIQGTTAFTAFNNTIYTLGAQDDIYETSYHNWYLDT---PFNHFNLAYKEGQYK--------DN--NNSWNKTVFP 155
porcine7          79 PIPITNNLPIQGTSTFTAFNNTIYSLGASDDLYETGYQNWYEDT---LWRSWYVAYKEGLVPKPTTATKEGV--GNSWSRLTLP 157
turkey2           79 MTPMTTQIAIQGTNTFTAFNNTIYALGYTDEMCETGFFNWYSS----EYDSPNLSLHEGLGK--------RH--DDTSYRYELP 148
pit_viper         79 PVPMTTQLAIQGSTVFTAFNNTIYSLGYTDTLYETDWFNWNSPA-SDHIDLLNLAYKEGLAR--------TV--NGTTTRYILP 151

cons              85   *:* ::.***:..*********:**  * : ** : **             :: :**                 :  :   * 168

                                      VR5                   VR6
murine_ChPV_NY   157 IYSWRTENARNASSSTYSY-LNG-------IDSYAVWPRTKD----KELIPTGVFWDPLNDANGILELRPGKNSMSFSWEQHPC 228
MKDV             157 IYSWRTENARNASSSTYSY-LNG-------IDSYAVWPRTKD----KELIPTGVFWDPLNDANGILELRPGKNSMSFSWEQHPC 228
rat2             158 VYYWKSPTPELNSVVTWTW-DQEYNSAAAGLIGNVAWPITGT---NTISLPDGVFWDPLTSPDDIMELRPGKNSMTWSWERHDC 237
Desmodus_rotond  156 VYSWRTANPRNVSDTTYSY-IPT-------VLSYSTWPRVAN----ESDIPNGLFWDPLTDPDSIMELRPGKNSMSFSWNAHDC 227
porcine7         158 TYLWSAPITAPETNWTWTW-NTNK-PY---PTAGTTWAHTDA-GTEQVVAPARCFWDPFTNTDSIQELRPGKNAMSFHWKTHAA 235
turkey2          149 IYLWPKPHVRVTSQRTMGDWPYN-------TEGYTTWPKNQQNNDDTNGIPTGLIWDPFNMPKMLQELRPGKNAISFSWHPHDC 225
pit_viper        152 TYLWRAQNARMASFDSWAT-EPN-------MSAYPVYPIGGKVSASEGEQPSGVFWDPFNRPNHITELRPGKNAIQYVWENHGC 227

cons             169  * *        :  :                .  .:.            *   :***:. .. : *******:: : *. * . 252

                                              VR7
murine_ChPV_NY   229 DENKWFNIDQIAKWFPYTVDTPYLNPQ---T-YGPPGSYKLY-GEDDPDQLTTPSSWT-AYSAKNDYTIPNLLDMPIVPMQWFW 306
MKDV             229 DENKWFNIDQIAKWFPYTVDTPYLNPQ---T-YGPPGSYKLY-GEDDPDQLTTPSSWT-AYSAKNDYTIPNLLDMPIVPMQWFW 306
rat2             238 DENKWYNIDQLARWAPYTADMPWVNLG---R-IGGAGSYQPD-DIEDPKSLSGPRYGT-N-PATEDYTIPNLANMPIVPVAWWW 314
Desmodus_rotond  228 DTNKWYNLDCIAKWTPYVHDNPFQHIG---R-AGQAGSYKIT-TTDDPDPLSTYSSWATTELTKDDFTIPNLLNMPVVPMNWWW 306
```

```
porcine7          236  DEHCWYNLDSLVKLFPYTPESGYSHNIREKTYQGPPGTRIVNENFQHPTPQTSISSDQ-S-KVFIEHDVPNILTAPSVPIHWFW  317
turkey2           226  DQNKWYNLDQIAWWWPYAAPSPYPNNN-------RPQTSQLS-TQCDPDRLQSKYE---N-TNPGDYTMPDMSLMPITPTGWWW  297
pit_viper         228  DDGIWFNMDHIASFSPYFGDHPWDSF----S-AGFPGSYKLNQNADDPEFLSPY-M-T-APTNKRNWTIPNLANMPIVPTGWFW  303

cons              253  *   *:*:* :.   **     :            . :        .*                 :  :*::   * .*  *:*  336

                                VR8                                 HI loop
murine_ChPV_NY    307  QEIQKSIAEVPD-----VKKPMLYWAGTEYECYKYGPTQCFLKGIPLFDDNDTHVATTTQGCFRISLHLAGKKRRSRIYAPTWG  385
MKDV              307  QEIQKSIAEVPD-----VKKPMLYWAGTEYECYKYGPTQCFLKGIPLFDDNDTHVATTTQGCFRISLHLAGKKRRSRIYAPTWG  385
rat2              315  IEMQKSIVGTYDDNSSPQKEEALYGPGTEYEQYKYPPTQCFIKGLPLFDDDGNHISTTTQGCFKVTLHLAAKKRRSRIYAPTWH  398
Desmodus_rotond   307  QEMQKSIAQTTT-----LKKPALFWAGTEYEQYKYPPTQCFLKGVPLFDDNGTHVETLTQGCFRVTLHLAAKKRRSRIFAPTWG  385
porcine7          318  IELERNLIEDKK-----VEKPQLGWPGTEWATPKYPPMNNFIKGIPLTDENETLVKTITMGCFRNGIHLSCKKRRSRMFAPTWG  396
turkey2           298  KEMQSSIILSPGEY--FSSRPDLFFAGTEYEFFKYPTMQHFIKIIPLFDSQGNHIDISANISVRTTLHLKCKKRRSAYFCPTYG  379
pit_viper         304  KEVKESIALTQDP----TTIPELMAAQTEYQQYKYPPTQWFIKGLPLFDAAGTHVETQTQGCVKITIHIQGKKRRSAIYAPTWG  383

cons              337   *:: .:               *  . **:   ** . : *:* :** *   . :   :  ..:  :*:  *****  :.**:   420

                             VR9                           VR10
murine_ChPV_NY    386  PLSWRQCYATDT---PFAPSMVRYRTGGARRTWTNINRDAEG----VHKDFHYREDPYDITSTVPDTRGTATLTDSKATMHP--  460
MKDV              386  PLSWRQCYATDT---PFAPSMVRYRTGGARRTWTNINRDAEG----VHKDFHYREDPYDITSTVPDTRGTATVTDSKATMHP--  460
rat2              399  PHTWRFTHTIHA---PRVGSYVRYRTGGARRTWTNLRAGP-G----LSGTIRVRSTPYNTNSTYATTATRTTPTTSTRMNY--  472
Desmodus_rotond   386  PLAWTQIYSIDA---PRAPNAVRYRTGGARRTWTNIEKES-G----E-SPYNIREDPYSTSTTYDRSITSNTETISKPQIR---  457
porcine7          397  PEM---THGINS---AFVLPPVRYRTGGARRSWQARTRDTRDK---EPQQTWYQWNPYMTG-TYSSTTTTSTYTTTTSRK----  466
turkey2           380  PLNWRMLYTNQPNRFNFRHSYVRYRTAGARLTWQNQAGTEETNPASTIDVSHPRTSPYIASSTN--TRMTTTFMTLEPRMSPVV  461
pit_viper         384  PFNWRHLYASDKQSRTFQNSYVRYRTGGARRTMTNYSLWP-T----PGSVDNPREPPYTTAKWTAATQTQGRVTQPPTSVTK--  460

cons              421  *      :  .          *****.*** :                     :  **        :          .        504


murine_ChPV_NY    461  -YEQAASGMYLNHKEMRQVRAAAEATR------------SQPAVA-MQTQ---------  496
MKDV              461  -YEQAASGMYLNHKEMRQVRAAAEATR------------SQPAVA-MQTQ---------  496
rat2              473  -----------------------------------------------------------  472
Desmodus_rotond   458  -YRQMVN--------------KRD-RT------------AETAVTMMDLQLPPNL----  484
porcine7          467  -----------------------------------------------------------  466
turkey2           462  TYDKES----------------GEATIREPPIATHRKARRPPNIP-CSTEEAMLISLDQ  503
pit_viper         461  -LVQALA------------------SR------------GKMATP--------------  474

cons              505                                                              563
```

B

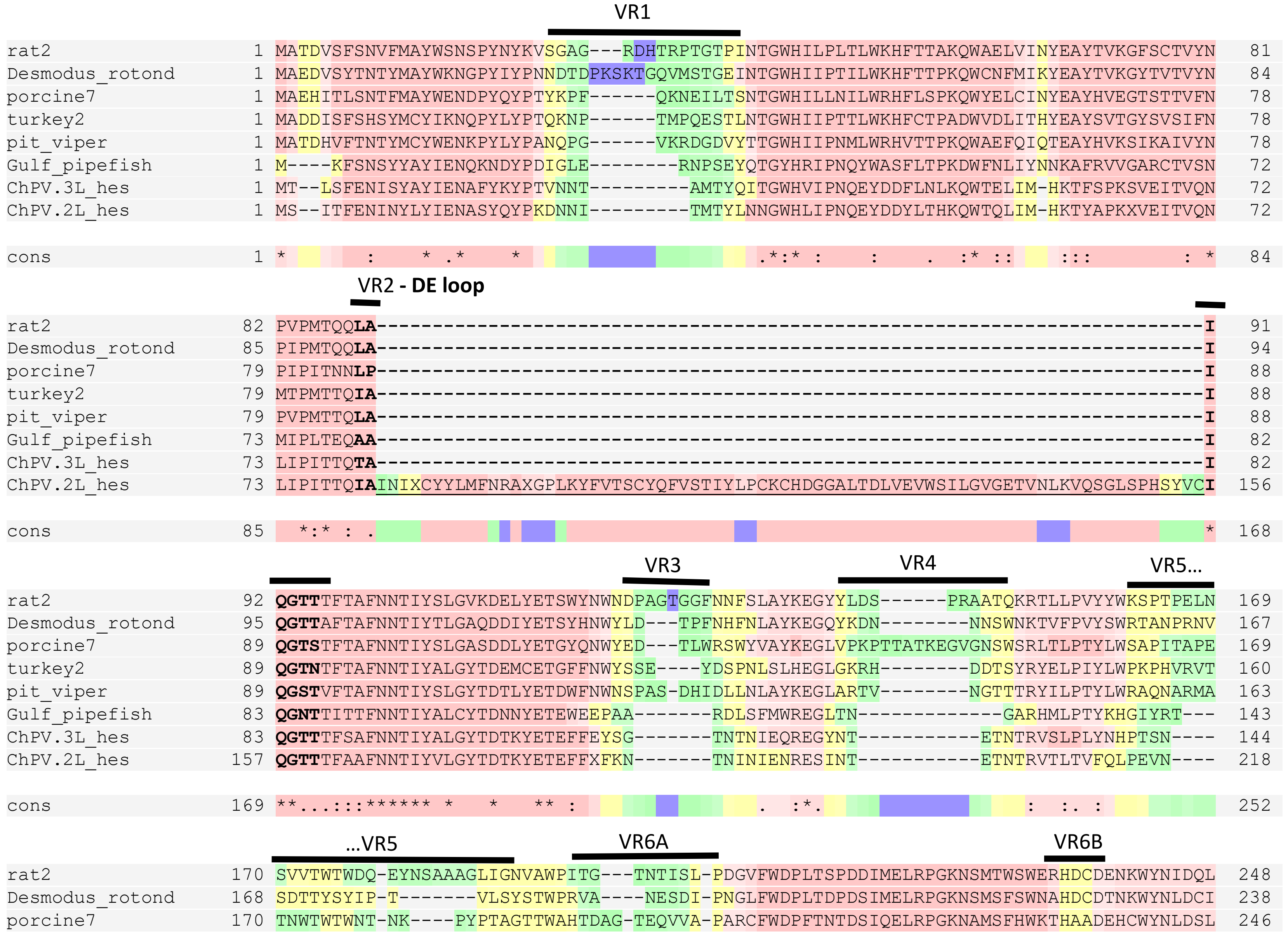
VR1
rat2 1 MATDVSFSNVFMAYWSNSPYNYKVSGAG---RDHTRPTGTPINTGWHILPLTLWKHFTTAKQWAELVINYEAYTVKGFSCTVYN 81
Desmodus_rotond 1 MAEDVSYTNTYMAYWKNGPYIYPNNDTDPKSKTGQVMSTGEINTGWHIIPTILWKHFTTPKQWCNFMIKYEAYTVKGYTVTVYN 84
porcine7 1 MAEHITLSNTFMAYWENDPYQYPTYKPF------QKNEILTSNTGWHILLNILWRHFLSPKQWYELCINYEAYHVEGTSTTVFN 78
turkey2 1 MADDISFSHSYMCYIKNQPYLYPTQKNP------TMPQESTLNTGWHIIPTTLWKHFCTPADWVDLITHYEAYSVTGYSVSIFN 78
pit_viper 1 MATDHVFTNTYMCYWENKPYLYPANQPG------VKRDGDVYTTGWHIIPNMLWRHVTTPKQWAEFQIQTEAYHVKSIKAIVYN 78
Gulf_pipefish 1 M----KFSNSYYAYIENQKNDYPDIGLE--------RNPSEYQTGYHRIPNQYWASFLTPKDWFNLIYNNKAFRVVGARCTVSN 72
ChPV.3L_hes 1 MT--LSFENISYAYIENAFYKYPTVNNT---------AMTYQITGWHVIPNQEYDDFLNLKQWTELIM-HKTFSPKSVEITVQN 72
ChPV.2L_hes 1 MS--ITFENINYLYIENASYQYPKDNNI---------TMTYLNNGWHLIPNQEYDDYLTHKQWTQLIM-HKTYAPKXVEITVQN 72
cons 1 * : * .* * .*:* : : . :* :: ::: : * 84
VR2 - DE loop
rat2 82 PVPMTQQLA--I 91
Desmodus_rotond 85 PIPMTQQLA--I 94
porcine7 79 PIPITNNLP--I 88
turkey2 79 MTPMTTQIA--I 88
pit_viper 79 PVPMTTQLA--I 88
Gulf_pipefish 73 MIPLTEQAA--I 82
ChPV.3L_hes 73 LIPITTQTA--I 82
ChPV.2L_hes 73 LIPITTQIAINIXCYYLMFNRAXGPLKYFVTSCYQFVSTIYLPCKCHDGGALTDLVEVWSILGVGETVNLKVQSGLSPHSYVCI 156
cons 85 *:* : . * 168
VR3
VR4
VR5...
rat2 92 QGTTTFTAFNNTIYSLGVKDELYETSWYNWNDPAGTGGFNNFSLAYKEGYYLDS------PRAATQKRTLLPVYYWKSPTPELN 169
Desmodus_rotond 95 QGTTAFTAFNNTIYTLGAQDDIYETSYHNWYLD---TPFNHFNLAYKEGQYKDN--------NNSWNKTVFPVYSWRTANPRNV 167
porcine7 89 QGTSTFTAFNNTIYSLGASDDLYETGYQNWYED---TLWRSWYVAYKEGLVPKPTTATKEGVGNSWSRLTLPTYLWSAPITAPE 169
turkey2 89 QGTNTFTAFNNTIYALGYTDEMCETGFFNWYSSE----YDSPNLSLHEGLGKRH--------DDTSYRYELPIYLWPKPHVRVT 160
pit_viper 89 QGSTVFTAFNNTIYSLGYTDTLYETDWFNWNSPAS-DHIDLLNLAYKEGLARTV--------NGTTTRYILPTYLWRAQNARMA 163
Gulf_pipefish 83 QGNTTITTFNNTIYALCYTDNNYETEWEEPAA-------RDLSFMWREGLTN-------------GARHMLPTYKHGIYRT--- 143
ChPV.3L_hes 83 QGTTTFSAFNNTIYALGYTDTKYETEFFEYSG-------TNTNIEQREGYNT-----------ETNTRVSLPLYNHPTSN---- 144
ChPV.2L_hes 157 QGTTTFAAFNNTIYVLGYTDTKYETEFFXFKN-------TNINIENRESINT-----------ETNTRVTLTVFQLPEVN---- 218
cons 169 **...:::****** * * ** : . :*. : :. : 252
...VR5
VR6A
VR6B
rat2 170 SVVTWTWDQ-EYNSAAAGLIGNVAWPITG---TNTISL-PDGVFWDPLTSPDDIMELRPGKNSMTWSWERHDCDENKWYNIDQL 248
Desmodus_rotond 168 SDTTYSYIP-T-------VLSYSTWPRVA----NESDI-PNGLFWDPLTDPDSIMELRPGKNSMSFSWNAHDCDTNKWYNLDCI 238
porcine7 170 TNWTWTWNT-NK----PYPTAGTTWAHTDAG-TEQVVA-PARCFWDPFTNTDSIQELRPGKNAMSFHWKTHAADEHCWYNLDSL 246

```
turkey2          161 SQRTMGDWPYN-------TEGYTTWPKNQQNNDDTNGI-PTGLIWDPFNMPKMLQELRPGKNAISFSWHPHDCDQNKWYNLDQI 236
pit_viper        164 SFDSWATEP-N-------MSAYPVYPIGGKVSASEGEQ-PSGVFWDPFNRPNHITELRPGKNAIQYVWENHGCDDGIWFNMDHI 238
Gulf_pipefish    144 ----------------------------------TTSQAHNVFYGWDPLCKAENILELRPGKNAVTFEWHA---KEEIWLNTHMM 191
ChPV.3L_hes      145 -------------------------------------N-KTFLFWDPLLNPNDIMELRPGKNAIKYSWHRSSADDHIVYNTSGL 190
ChPV.2L_hes      219 -------------------------------------N-KTMLYWGPLINPSEIMELRPGKNAIKYTWHRV-YDELDHRNL--- 260

cons             253                                             *.*:  .. : *******:: : *.    .     *     336

                                   VR7
rat2             249 ARWAPYTAD-MPWVNLGRI----GGAGSYQPD-DIEDPKSLSGP-RYG-TNPATEDY-TIPNLANMPIVPVAWWIEMQKSIVG 323
Desmodus_rotond  239 AKWTPYVHD-NPFQHIGRA----GQAGSYKIT-TTDDPDPLSTYSSWATTELTKDDF-TIPNLLNMPVVPMNWWWQEMQKSIAQ 315
porcine7         247 VKLFPYTPE-SGYSHNIREKTYQGPPGTRIVNENFQHPTPQTSISSDQ--SKVFIEH-DVPNILTAPSVPIHWFWIELERNLIE 326
turkey2          237 AWWWPYAAP-SPYPNNN-------RPQTSQLSTQC-DPDRLQSK--YE--NTNPGDY-TMPDMSLMPITPTGWWWKEMQSSIIL 306
pit_viper        239 ASFSPYFGD-HPWDSFS-A----GFPGSYKLNQNADDPEFLS-P--YMTAPTNKRNW-TIPNLANMPIVPTGWFWKEVKESIAL 312
Gulf_pipefish    192 QQFDPTHTP-GI-ANTA---------TVY------------------------SQEIHNQTHSNVTPHSHLNRWISQTDTAPAT 240
ChPV.3L_hes      191 FTLDPTFNSENMF----------------------------------------LMDF-DRRNTNVTPYQQHQHWLKSNHTGFGQ 233
ChPV.2L_hes      261 -NITP------------------------------------------------------------YLHHQHWIHRNQTEFGR 281

cons             337     *                                                                    :    .      420

                       VR8                          HI-loop                                    VR9...
rat2             324 TYDDNSSPQKEEALYGPGTEYEQYKYPPTQCFIKGLPLFDDDGNHISTTTQGCFKVTLHLAAKKRRSRIYAPTWHPHTWR-FTH 406
Desmodus_rotond  316 TTT-----LKKPALFWAGTEYEQYKYPPTQCFLKGVPLFDDNGTHVETLTQGCFRVTLHLAAKKRRSRIFAPTWGPLAWT-QIY 393
porcine7         327 DKK-----VEKPQLGWPGTEWATPKYPPMNNFIKGIPLTDENETLVKTITMGCFRNGIHLSCKKRRSRMFAPTWGPEM----TH 401
turkey2          307 SPGE--YFSSRPDLFFAGTEYEFFKYPTMQHFIKIIPLFDSQGNHIDISANISVRTTLHLKCKKRRSAYFCPTYGPLNWR-MLY 387
pit_viper        313 TQD----PTTIPELMAAQTEYQQYKYPPTQWFIKGLPLFDAAGTHVETQTQGCVKITIHIQGKKRRSAIYAPTWGPFNWR-HLY 391
Gulf_pipefish    241 ARH-----WSKQAIQRPGIMEQQFRYPIPNWFIKMIPLFDSQNNLIKTTAQVLITMELTVDTIPQSLAINMPIIDDII------ 314
ChPV.3L_hes      234 ----------HFQYQMSGLYQNNFTKPIPNFFIKMIPLFDENNALINTTAQICIHKKITFDVTPLDASLNRPIIPSMLYDNEFY 307
ChPV.2L_hes      282 ----------QFNYQMSGMPENNFFKSIPNMFIKMIPLFDENNALINTTAQVCIHRKITFDIEPLFSSINRPLAPENMYNTPLY 355

cons             421                 .         .  : *:* :** *     :.  :   .   : .           *            504

                      ...VR9                                             VR10
rat2             407 TIHAP---RVGSYVR-----------------YRTGGARRTWTNLRAGP--GLSG---TIRVRSTPYNTNSTYATTATTRTT-- 463
Desmodus_rotond  394 SIDAP---RAPNAVR-----------------YRTGGARRTWTNIEKES--G-ES---PYNIREDPYSTSTTYDRSITSNTE-- 449
porcine7         402 GINSA---FVLPPVR-----------------YRTGGARRSWQARTRDT--RDKEPQ-QTWYQWNPYMTGT-YSSTTTTSTY-- 459
turkey2          388 TNQPNRFNFRHSYVR-----------------YRTAGARLTWQNQAGTEETNPASTIDVSHPRTSPYIASSTNTRMTTTFMTLE 454
pit_viper        392 ASDKQSRTFQNSYVR-----------------YRTGGARRTMTNYSLW--PTPGS---VDNPREPPYTTAKWTAATQTQGRVTQ 453
Gulf_pipefish    315 ---------------------------------------------A--PNH-------TPNSVPYCMFRY-QPIIPINVG-- 338
ChPV.3L_hes      308 TTNYKGITFH-AHAAYQHNIGKLQDMTAKQKEKKINGKILTWPIYYEAP----KD---KIKNDFFPWVTQTERNNELI-HLG-- 380
ChPV.2L_hes      356 TQKY----------------------------------------------------------------NLYQSLSIYA---------- 369
```

```
cons               505  .                                      :         588

rat2               464 PTTSTRMNY---------------------------------------- 472
Desmodus_rotond    450 TISKPQIRYRQMVNKRDRTAETAVTMMDLQLPPNL-------------- 484
porcine7           460 TTTTSRK------------------------------------------ 466
turkey2            455 PRMSPVVTYDKESGEATIREPPIATHRKARRPPNIPCSTEEAMLISLDQ 503
pit_viper          454 PP----TSVTKLVQ-------ALASRGKMATP----------------- 474
Gulf_pipefish      339 VLPAPPTKGVF-IP-------ELPARSDPTSD------------VE--- 364
ChPV.3L_hes        381 IVTKTK------------------------------------------- 386
ChPV.2L_hes        370 ------------------------------------------------- 369

cons               589                                                   637
```

Figure S3

Variable regions at the derived amino acid sequence level identified among chapparvovirus capsid proteins. (A) Alignment of amniote chapparvovirus capsid protein ORF (VP) derived amino acid sequences, containing both isolates of murine origin. Variable regions (VRs) are marked by the black bars and coloring is based on sequence similarity (red = highly similar, blue = not similar). The conserved loops making up the fivefold symmetry axes of the capsid are highlighted in bold. (B) The same alignment incorporating the complete VP protein sequences of all novel chapparvovirus sequences reported in this study.
